# Fall Risk Prediction for Community-Dwelling Older Adults: Analysis of Assessment Scale and Evaluation Items without Actual Measurement

**DOI:** 10.3390/ijerph21020224

**Published:** 2024-02-14

**Authors:** Akihiko Murayama, Daisuke Higuchi, Kosuke Saida, Shigeya Tanaka, Tomoyuki Shinohara

**Affiliations:** 1 Department of Physical Therapy, Faculty of Rehabilitation, Gunma University of Health and Welfare, Maebashi Plaza Genki 21 6-7F, 2-12-1 Hon-machi, Maebashi-shi 371-0023, Japan; 2 Department of Physical Therapy, Faculty of Health Care, Takasaki University of Health and Welfare, 501 Naka Orui-machi, Takasaki-shi 370-0033, Japan; higuchi-d@takasaki-u.ac.jp (D.H.); saida@takasaki-u.ac.jp (K.S.); tanaka-s@takasaki-u.ac.jp (S.T.); shinohara-t@takasaki-u.ac.jp (T.S.)

**Keywords:** coronavirus disease, community-dwelling older adults, falls, Frailty Screening Index, Questionnaire for Medical Checkup of Old-Old

## Abstract

The frequency of falls increases with age. In Japan, the population is aging rapidly, and fall prevention measures are an urgent issue. However, assessing fall risk during the coronavirus disease pandemic was complicated by the social distancing measures implemented to prevent the disease, while traditional assessments that involve actual measurements are complicated. This prospective cohort study predicted the risk of falls in community-dwelling older adults using an assessment method that does not require actual measurements. A survey was conducted among 434 community-dwelling older adults to obtain data regarding baseline attributes (age, sex, living with family, use of long-term care insurance, and multimorbidity), Frailty Screening Index (FSI) score, and Questionnaire for Medical Checkup of Old-Old (QMCOO) score. The participants were categorized into fall (*n* = 78) and non-fall (*n* = 356) groups. The binomial logistic regression analysis showed that it is better to focus on the QMCOO sub-item score, which focuses on multiple factors. The items significantly associated with falls were Q5 (odds ratio [OR] 1.95), Q8 (OR 2.33), and Q10 (OR 3.68). Our results were similar to common risk factors for falls in normal times. During the pandemic, being able to gauge the risk factors for falls without actually measuring them was important.

## 1. Introduction

In Japan, individuals aged ≥65 years are defined as older adults. As of 17 September 2023, Japan’s older adult population was 36.23 million people, accounting for 29.1% of the total population, the highest ever. Additionally, the number of people aged ≥75 years exceeded 20 million for the first time; 1 in 10 people is over 80 years old. Furthermore, Japan has the highest percentage of older adults worldwide (of the 200 countries/regions) [1].

The coronavirus disease 2019 (COVID-19), which has been circulating around the world since 2020, has had a significant impact on the lives of the Japanese people. The observance of social distancing to prevent infectious diseases and the associated self-restraint continued for approximately 3 years. COVID-19 has presented challenges for both the increased risk of adverse outcomes for older adults if infected and the decreased quality of life due to loneliness [2]. Community-dwelling older adults in Japan are reporting muscle weakness because they were unable to go out during the COVID-19 outbreak [3]. There were concerns that these changes in the social activities of the older adults could lead to new onset or worsening of frailty. There was also concern about “corona frailty”, which could be considered a secondary effect of COVID-19 [4]. A pre-pandemic meta-analysis reported that the frailty rate in Japan was 7.5% for frailty and 48.1% for pre-frailty [5]. Conversely, it has been pointed out that frailty and pre-frailty tend to increase among older adults who have been forced to change their social activities due to the spread of COVID-19 [6].

A systematic review and meta-analysis of frailty and falls in older adults found that older adults with frailty were 1.8 times more likely to fall than robust older adults [7]. Moreover, a 10-year follow-up study of women aged ≥75 years found that the presence of frailty at baseline was a significant risk for the occurrence of falls [8]. Compared to pre-pandemic times, the number of respondents reporting fear of falling or not being able to walk in the future increased significantly. Older adults are encouraged to stay at home, which increases their risk of living a sedentary lifestyle and developing chronic diseases [9]. Therefore, there are concerns that older adults may be at increased risk of falling due to the pandemic, which stopped them from going out and reduced their physical activity [10]. The pandemic has led to a rapid shift in the delivery of health services from face-to-face interactions to remote medical care and hospital care [11]. The pandemic has significantly increased the uptake of telemedicine. However, the effectiveness of remote fall prevention is unclear [12].

In Japan, preventive measures against the spread of COVID-19 are still emphasized; however, a balance is needed between maintaining pre-pandemic daily life and taking measures to prevent infection. Data from countries that lifted restrictions earlier than Japan showed that fall rates were lower at the beginning of the pandemic compared to the pre-pandemic levels; however, once movement restrictions were lifted, falls increased [13].

Implementing such measures requires accurate fall risk assessment using predictive methods [14]. In situations where the spread of COVID-19 is considered, non-face-to-face evaluation methods that do not require actual measurement are used instead of the conventional methods that require actual face-to-face measurement. However, to date, no reliable tool for predicting the risk of falls using an assessment method that does not require actual measurements is available. Even if COVID-19 subsides, those who have concerns about face-to-face assessments should be considered.

In Japan, concerns about the health of older adults have only intensified as the pandemic has made it impossible to provide local frailty prevention support. Therefore, based on discussions with local welfare commissioners who support older adults, we are continuing to conduct frailty prevention awareness, self-checks, and surveys on the actual state of frailty, along with monitoring activities that adhere to social distancing [15]. This study is a sub-analysis of a series of cohort studies conducted by a group of researchers focusing on evaluations that contribute to the prediction of falls. Furthermore, we cannot disregard the World Health Organization’s (WHO) emphasis on clarifying and implementing effective interventions based on reality [16]. Additionally, the WHO points out the need to prepare for other pathogens (Disease X) that can cause future epidemics and pandemics [17].

Given the above-mentioned points, we developed a fall risk assessment method that does not require actual measurements in the community. This study aimed to examine useful indicators for predicting falls in community-dwelling older adults during the COVID-19 pandemic, and we conducted a survey using a questionnaire. We hypothesized that there would be knowledge that could contribute to the extraction of risk factors for falls during the pandemic, even from evaluations that do not involve actual measurements and analysis of evaluation items. By verifying this hypothesis, we hope to help prepare for future emergencies.

## 2. Materials and Methods

### 2.1. Study Design and Participants

This prospective cohort study was conducted among community-dwelling older adults. The participants were people aged ≥65 years living in Takasaki City, Gunma Prefecture. The population of Takasaki City is approximately 370,000 people, and the aging rate is approximately 28.1%. Takasaki City is a core city, and the aging rate is almost the same as the national average. In Takasaki City, “Kayoi no Ba activities (Resident-based care prevention activities)” have been reduced or canceled since the state of emergency was declared on 16 April 2020. This continued until 8 May 2023, when COVID-19 became a “Class 5 infectious disease: Until hospitalization and movement restrictions based on laws and regulations are lifted”. During this period, preventive care activities by physical therapists, occupational therapists, speech therapists, and others, which were held approximately 250 times a year at salons across Takasaki City, were reduced or canceled. Although the area was actively promoting preventive care, there were concerns that residents’ functions would deteriorate as the above-mentioned activities were curtailed. Therefore, with the cooperation of local welfare commissioners and others, we decided to distribute questionnaires not only to those who participated in the above activities but also to older adults living in the targeted areas.

**Figure 1:**
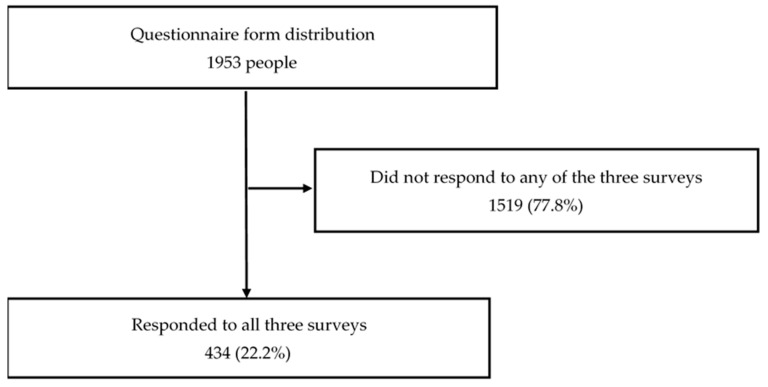
Flowchart of the participant selection process.

First, a baseline survey was conducted among 1953 community-dwelling older adults. A questionnaire was distributed to 1279 people who responded, and 434 (age: 80.0 ± 6.0 [range: 66–98] years) who were followed up from the baseline to the third survey were selected (Figure 1). The baseline survey was conducted from 11 May 2020 to 10 July 2020, the secondary survey was conducted from 11 November 2020 to 10 January 2021, and the tertiary survey was conducted from 11 May 2021 to 10 July 2021. Owing to the increased risk of the spread of COVID-19 during the survey period, survey items that did not involve actual measurements were adopted. Civil welfare commissioners who regularly visited the homes of study participants distributed questionnaires and research instructions to prospective participants at 6-month intervals. Individuals who were willing to participate in this study were asked to return the questionnaires and research participation consent form by mail. The distribution did not necessarily require a face-to-face meeting; instead, survey materials were mailed, and explanations and safety confirmations were conducted over the phone. By having participants mail the questionnaires, research team members were able to track who answered and returned the questionnaires and then aggregated the data. A digest of these aggregated results was made into a booklet to help share information with distributors.

This study complied with the Declaration of Helsinki and was planned based on the “Ethical Guidelines for Medical Science Research Involving Human Subjects”. It was approved by the Research Ethics Committee of Takasaki University of Health and Welfare (approval number: 2009) and was registered with the University Hospital Medical Information Network (UMIN000040335). The questionnaire, a document indicating the purpose and content of this study, and contact points for inquiries were distributed to the research participants. Writing of their names on the questionnaire confirmed the participants’ consent to participate in this study.

### 2.2. Measurements

The survey items included the baseline attributes (age, sex, living with family, use of long-term care insurance, and multimorbidity), Frailty Screening Index (FSI) score [18], and Questionnaire for Medical Checkup of Old-Old (QMCOO) score [19]. Based on the WHO criteria, multimorbidity was defined as the presence of two or more chronic diseases [20]. The survey questionnaire was designed to be completed in approximately 10 min. Given the perceived time burden of responding, data on participants’ education level, socioeconomic status/income level, and marital status were not required.

The FSI measurement instrument was administered in all surveys. It is a self-administered questionnaire with yes/no answers [18] to the following five questions: (1) “Have you lost 2 kg or more in the past 6 months? (2) Do you think you walk slower than before? (3) Do you go for a walk for your health at least once a week? (4) Can you recall what happened 5 min ago? and (5) In the past 2 weeks, have you felt tired without a reason?” with each item rated based on the question raised and criteria provided. A score of ≥3 points indicates frailty, while a score of 1–2 indicates pre-frailty. The FSI is widely used to investigate frailty in community-dwelling older adults and to assess the changes in frailty status over time. Additionally, it can accurately determine the presence of frailty (a phenotype model) in older Japanese individuals [21]. The Japanese version of the Cardiovascular Health Study criteria (revised J-CHS criteria) [22], which includes measurements of walking speed and grip strength, is widely used to evaluate frailty in Japan. However, this time, we adopted FSI, which does not require actual measurements and is evaluated using a questionnaire.

The QMCOO was administered during the first and second surveys. It comprises the following 15 questions: (1) How is your current health condition? (2) Are you satisfied with your daily life? (3) Do you regularly eat three meals a day? (4) Compared to 6 months ago, do you find it more difficult to eat tough or solid foods? (5) Do you find yourself choking on tea or soup? (6) Have you lost 2–3 kg or more in the past 6 months? (7) Do you think that your walking speed has slowed down compared with that before? (8) Have you fallen in the past year? (9) Do you exercise (such as taking walks) at least once a week? (10) Do people around you comment on your forgetfulness? For example, whether they tell you, “You are always asking the same thing”. (11) Are there times when you do not remember today’s date? (12) Do you smoke? (13) Do you go out at least once a week? (14) Do you regularly meet with your family or friends? and (15) When you are not feeling well, do you have someone close to talk to? [19]. It comprises the following 10 dimensions: health, mental health, eating habits, oral function, weight change, exercise/fall, cognitive function, smoking, social participation, and social support. Previous research has proposed a method of scoring the 15 items of QMCOO with a score of 0 or 1, and frailty is defined by a QMCOO score of ≥4 points [23]. In Japan, the Kihon Checklist is sometimes used as a useful tool for frailty screening [24]. However, since there were 25 questions, considering the burden of answering, we adopted QMCOO, which has a smaller number of questions. Additionally, QMCOO interviews were not conducted in the third survey due to concerns about a decline in the response rate.

The fall group was defined as individuals who fell at least once between the conductance of the three surveys. Their baseline attributes, baseline FSI scores, and QMCOO scores were compared with those of the non-fall group (individuals who did not fall). The FSI score was calculated according to the method described by Yamada et al. [18], while the QMCOO score was calculated according to the method described by Shinohara et al. [23].

### 2.3. Statistical Analyses

We calculated the required sample size for interval estimation of population proportions in this questionnaire survey. The error was set at 5%, and the confidence level was set at 90%. Since it was difficult to predict the population proportion, the predicted population proportion was set at 50%. The required sample size in this case was 385. There was missing data, and for this reason, the raw data were preprocessed in Microsoft Excel and imported to EZR [25] (EZR on R commander ver. 1.61). First, the R package naniar was used to check missing value information [26]. If excluding missing values would result in selection bias [27], we decided to impute missing values using the multiple imputation method R package mice [28,29], which primarily focuses on imputing missing values in multivariate data [30]. We confirmed that the exclusion of missing values caused a selection bias in the data using the R package naniar [26]. Therefore, the missing values were imputed using the multiple imputation method (R package, mice [28,29]). It is necessary to specify the number of pseudo-complete data m and the substitution method for the R package mice. In this study, m was set to 10, and the method was set to predictive mean matching (PMM). PMM combines regression substitution and matching to randomly select and substitute observed values close to the regressed values, and the results were summarized using the standard Rubin’s rule [31]. Next, the Shapiro–Wilk test was used to assess the normality of the data. Normality was not observed in either the fall or non-fall group. Therefore, the Mann–Whitney U test was used to assess for any differences in age, FSI score, and QMCOO score. The chi-square test was used to test for any differences in sex, living with family members, and the ratio of multimorbidity. However, Fisher’s exact test was used for participants whose expected scores in each of the cells were <5. Odds ratios and 95% confidence intervals were determined using Binomial logistic regression analysis (forced entry). The presence or absence of falls was used as the dependent variable, and items with significant differences between groups were used as independent variables. Moreover, when performing forced entry, we checked the variance inflation factor and selected only those items for which no multicollinearity was observed. Independent predictors were identified through multivariate analysis. Furthermore, the “one in ten” rule regarding the predictive reliability of each factor was emphasized [32,33,34]. According to this rule, at least 10 events are required for each included predictor. In this case, at least 10 participants for each predictor were included. If this rule of thumb is not met, this domain is automatically rated as having a high risk of bias [35]. Statistical analyses were performed using EZR [25] (EZR on R commander ver. 1.61), with a significance level of 5%.

## 3. Results

Overall, 434 participants met the inclusion criteria. There were 78 and 356 patients in the fall and non-fall groups, respectively. The fall group had significantly higher use of the nursing care insurance system (*p* < 0.05), FSI (*p* < 0.01), and QMCOO scores (*p* < 0.0001) (Table 1). Significant differences between the groups were observed for the following FSI sub-items: Q2 (*p* = 0.008) and Q5 (*p* < 0.001) (Table 2). Significant differences between the groups were observed for the following QMCOO sub-items: Q1 (*p* < 0.001), Q5 (*p* = 0.001), Q7 (*p* = 0.008), Q8 (*p* < 0.0001), Q10 (*p* < 0.0001), and Q14 (*p* = 0.015) (Table 3).

We used binomial logistic regression analysis (adjusted according to age, sex, living with family, use of nursing care insurance system, and previous fall history as Q8 of QMCOO) with the FSI sub-items with significant differences that were forcibly input. Consequently, no significant variables were extracted (Table 4). Similarly, we used binomial logistic regression analysis (adjusted according to age, sex, living with family, and use of nursing care insurance system) with the QMCOO sub-items with significant differences that were forcibly input. Thus, three variables (Q5, 8, and 10) were extracted (Table 5). The result of the model χ^2^ test yielded a *p*-value of <0.00001, while that of Hosmer and Lemeshow’s test yielded a *p*-value of 0.517, indicating a good fit for the model.

## 4. Discussion

A decline in not only physical function but also cognitive function increases the risk of falls. Repeated falls not only make the lifestyle of older adults more dependent but also impair their quality of life and increase society’s medical costs; therefore, strategies are needed to prevent repeated falls among older adults [36].

The authors hypothesized that there would be knowledge that could contribute to the extraction of risk factors for falls during the pandemic, even from evaluations that do not involve actual measurements and analysis of evaluation items. By verifying this hypothesis, we hope to help prepare for future emergencies. Univariate analysis revealed that the fall group had significantly higher FSI and QMCOO scores than the non-fall group. However, the results of the binomial logistic regression analysis showed that it is better to focus on the scores of the QMCOO sub-items, which encompass multiple factors, such as physical function, cognitive function, and changes in other aspects, than the FSI sub-items. Our study showed that “score1” responses to the QMCOO sub-items (Q5, 8, and 10) revealed the factors significantly associated with falls. These questions evaluated an individual’s decreased swallowing function, past fall history, and subjective cognitive decline, which were identified to be significantly associated with future fall events. Although the QMCOO is not primarily a rating scale for predicting falls, our results suggest that various aspects of the QMCOO may make it an advantageous tool. Our results were similar to common risk factors for falls in normal times. During the pandemic, it is important to be able to gauge the risk factors for falls without actually measuring them. Conditions associated with a decrease in swallowing function, including mild choking, are reported to be associated with a decrease in physical function, such as walking speed and grip strength [37]. This finding suggests that systemic weakness progresses in parallel with diminished swallowing function [38]. Regardless of the presence or absence of objective decline in cognitive function, it has been pointed out that subjective decline in cognitive function affects falls in community-dwelling older adults. In other words, a correlation between subjective cognitive decline and falls has been reported [39]. A history of past falls is well-known as an independent risk factor for falls [40].

Furthermore, effective multifaceted interventions aimed at preventing falls in older adults should be based on individual fall risk factors rather than general interventions that ignore individual characteristics. In the guidelines, plans to prevent falls and related injuries should incorporate older people’s goals, values, and preferences [41]. Three years have passed since the global outbreak of COVID-19, and although fewer people seem concerned about the disease than before, some community-dwelling older adults may continue being anxious about face-to-face assessments. Therefore, improving the accuracy of non-face-to-face fall prediction assessments is meaningful. Future suggestions could include comparing survey data with more intensive fall assessment in clinical settings to ensure that this is a reliable method when clinical assessment is not available for risk assessment.

This study has some limitations. First, the survey response rate was only 22.2%; thus, we could not investigate and analyze the reasons for the participants’ inability to respond to the survey. Second, the sample size differed considerably between the two groups. Because the number of falls is self-reported, oversight bias may exist. Hence, future studies should verify the external validity of this questionnaire-based survey in a larger population. Although these results could be considered as a starting point for screening when assessing fall risk, the results are very limited. Third, occasional falls can be accidental and are usually related to external factors. Conversely, recurrent falls in older adults are generally associated with multifactorial intrinsic factors, suggesting a more complex risk model [42]. It is also necessary to consider these points. Despite these limitations, this cohort study is considered valuable as it was conducted in the early stages of the COVID-19 pandemic in Japan. The results of this study may contribute to the development of a new method for fall risk assessment in addition to that used for conventional frailty assessment. Our method enables continuous assessment without the need to perform complicated procedures with the help of family members or volunteers who are not specialists in fall and frailty prevention. Therefore, in situations where the intervention of others cannot be expected, older adults need to recognize their own issues and take action as much as possible. Therefore, it may be useful from the perspective of fostering health literacy during normal times.

## 5. Conclusions

This study aimed to predict the risk of falls among community-dwelling older adults using an assessment method that does not require actual measurements. These fall-related factors may require further validation in the future, as few studies have reported a causal relationship with falls since before the pandemic. Furthermore, in situations where intervention from others is not possible, older adults need to recognize their own issues and take as much action as possible. Thus, this may foster health literacy during normal times. As our results of risk factors were similar to those observed normally, our findings are useful. Additionally, during times such as pandemics, it is important to be able to measure risk factors without accurate measurements owing to restrictions in place.

## Figures and Tables

**Table 1:** Group comparisons of age, sex, living arrangement, multimorbidity, use of nursing care insurance system, and total scores of FSI and QMCOO.

	Overall	Fall Group	Non-Fall Group	*p*-Value
	(*n* = 434)	(*n* = 78)	(*n* = 356)	
Age, years, median (interquartile range)	80	81	79	0.130
	(75–84)	(75–85)	(75–84)	
Sex (female), *n* (%)	339 (78.1)	60 (76.9)	279 (78.4)	0.764
Living with family (alone), *n* (%)	339 (78.1)	63 (80.8)	276 (77.5)	0.634
Multimorbidity (applicable), *n* (%)	203 (46.8)	41 (52.6)	162 (45.5)	0.263
Use of nursing care insurance system (Yes), *n* (%)	82 (18.9)	22 (28.2)	60 (16.9)	0.025
FSI (score), median (interquartile range)	1	1	1	<0.01
	(0–2)	(1–2)	(0–2)	
QMCOO (score), median (interquartile range)	2	3	2	<0.0001
	(1–4)	(2–5)	(1–3)	

Age, FSI score, and QMCOO score: Mann–Whitney U test. Sex, living with family, multimorbidity, and use of nursing care insurance system: Chi-square test. FSI, Frailty Screening Index; QMCOO, Questionnaire for Medical Checkup of Old-Old.

**Table 2:** Group comparisons of the scores for the FSI sub-items.

FSI Sub-Items	Overall (*n* = 434)	Fall Group (*n* = 78)	Non-Fall Group (*n* = 356)	*p*-Value
Q1 (score: 1), *n* (%)	37 (8.5)	7 (9.0)	30 (8.4)	0.825
Q2 (score: 1), *n* (%)	208 (47.9)	48 (61.5)	160 (44.9)	0.008
Q3 (score: 1), *n* (%)	119 (27.4)	26 (33.3)	293 (26.1)	0.209
Q4 (score: 1), *n* (%)	23 (5.30)	6 (7.70)	17 (4.80)	0.275
Q5 (score: 1), *n* (%)	69 (15.9)	23 (29.5)	46 (12.9)	<0.001

The sub-items of the frailty screening index: Q1 and 4: Fisher’s exact test; Q2, 3, and 5: chi-square test. FSI, Frailty Screening Index.

**Table 3:** Group comparisons of scores for the QMCOO sub-items.

QMCOO Sub-Items	Overall (*n* = 434)	Fall Group (*n* = 78)	Non-Fall Group (*n* = 356)	*p*-Value
Q1 (score: 1), *n* (%)	39 (9.0)	16 (20.5)	23 (6.5)	<0.001
Q2 (score: 1), *n* (%)	55 (12.7)	11 (14.1)	44 (12.4)	0.707
Q3 (score: 1), *n* (%)	21 (4.8)	5 (6.4)	16 (4.5)	0.558
Q4 (score: 1), *n* (%)	129(29.7)	29 (37.2)	100 (28.1)	0.132
Q5 (score: 1), *n* (%)	94(21.7)	28(35.9)	66(18.5)	0.001
Q6 (score: 1), *n* (%)	37 (8.5)	7 (9.0)	30 (8.4)	0.825
Q7 (score: 1), *n* (%)	208 (47.9)	48 (61.5)	160 (44.9)	0.008
Q8 (score: 1), *n* (%)	103 (23.7)	34 (43.6)	69 (19.4)	<0.0001
Q9 (score: 1), *n* (%)	119 (27.4)	26 (33.3)	93 (26.1)	0.209
Q10 (score: 1), *n* (%)	33 (7.6)	16 (20.5)	17 (4.8)	<0.0001
Q11 (score: 1), *n* (%)	78 (18.0)	20 (25.6)	58 (16.3)	0.071
Q12 (score: 1), *n* (%)	60 (13.8)	9 (11.5)	51 (14.3)	0.591
Q13 (score: 1), *n* (%)	31(7.1)	6 (7.7)	25 (7.0)	0.809
Q14 (score: 1), *n* (%)	20(4.6)	8(10.3)	12(3.4)	0.015
Q15 (score: 1), *n* (%)	17(3.9)	4(5.1)	13(3.7)	0.522

The sub-items of QMCOO: Q1, 2, 4, 5, 7–12, and 14: chi-square test; Q3, 6, 13, and 15: Fisher’s exact test. QMCOO, Questionnaire for Medical Checkup of Old-Old.

**Table 4:** Results of binominal logistic regression analysis of scores for the FSI sub-items.

FSI Sub-Items	Odds Ratio	95% Confidence Interval	*p*-Value
Q2 (score 1)	1.42	0.82–2.46	0.210
Q5 (score 1)	1.84	0.97–3.47	0.059

Binomial logistic regression analysis was performed after the two items with significant differences were forcibly input and adjusted according to age, sex, living with family, use of nursing care insurance system, and previous fall history as Q8 of QMCOO. FSI, Frailty Screening Index; QMCOO, Questionnaire for Medical Checkup of Old-Old.

**Table 5:** Results of binominal logistic regression analysis of scores for the QMCOO sub-items.

QMCOO Sub-Items	Partial Regression Coefficient	Odds Ratio	95% Confidence Interval	*p*-Value
Q1 (score 1)		1.85	0.80–4.26	0.146
Q5 (score 1)	0.666	1.95	1.08–3.51	0.026
Q7 (score 1)		1.15	0.63–2.09	0.640
Q8 (score 1)	0.846	2.33	1.31–4.16	0.004
Q10 (score 1)	1.303	3.68	1.59–8.51	0.002
Q14 (score 1)		2.78	0.96–8.06	0.059

Binomial logistic regression analysis was performed after the six items with significant differences were forcibly input and adjusted according to age, sex, living with family, and use of the nursing care insurance system. QMCOO, Questionnaire for Medical Checkup of Old-Old.

## Data Availability

The data that support the findings of this study are available from the corresponding author [A.M.] upon reasonable request.
